# Neurochemical Profile of *BRAF*^V600E^/*Akt*^T308D/S473D^ Mouse Gangliogliomas Reveals Impaired GABAergic System Inhibition

**DOI:** 10.1159/000528587

**Published:** 2022-12-20

**Authors:** Maria Kyriazi, Philipp Müller, Julika Pitsch, Karen M.J. van Loo, Anne Quatraccioni, Thoralf Opitz, Susanne Schoch, Albert J. Becker, Silvia Cases-Cunillera

**Affiliations:** ^a^Institute of Neuropathology, Section for Translational Epilepsy Research, Medical Faculty, University of Bonn, Bonn, Germany; ^b^Department of Epileptology, Medical Faculty, University of Bonn, Bonn, Germany; ^c^Department of Epileptology, Neurology, RWTH Aachen University, Aachen, Germany; ^d^Institute of Experimental Epileptology and Cognition Research, Medical Faculty, University of Bonn, Bonn, Germany

**Keywords:** Ganglioglioma, Neurochemical, GABAergic, Interneurons, In utero electroporation

## Abstract

Gangliogliomas (GGs), composed of dysmorphic neurons and neoplastic astroglia, represent the most frequent tumor entity associated with chronic recurrent epileptic seizures. So far, a systematic analysis of potential differences in neurochemical profiles of dysmorphic tumoral neurons as well as neurons of the peritumoral microenvironment (PTME) was hampered by the inability to unequivocally differentiate between the distinct neuronal components in human GG biopsies. Here, we have applied a novel GG mouse model that allows to clearly resolve the neurochemical profiles of GG-intrinsic versus PTME neurons. For this purpose, glioneuronal tumors in mice were induced by intraventricular in utero electroporation (IUE) of piggyBac-based plasmids for *BRAF*<sup>*V600E*</sup> and activated Akt (*Akt*<sup>*T308D/S473D*</sup>, further referred to as *Akt*<sup>*DD*</sup>) and analyzed neurochemically by immunocytochemistry against specific marker proteins. IUE of *BRAF*<sup>*V600E*</sup>/*Akt*<sup>*DD*</sup> in mice resulted in tumors with the morphological features of human GGs. Our immunocytochemical analysis revealed a strong reduction of GABA_A_Rα1 immunoreactivity in the tumor compared to the PTME. In contrast, the extent of NMDAR1 immunoreactivity in the tumor appeared comparable to the PTME. Interestingly, tumor cells maintained the potential to express both receptors. Fittingly, the abundance of the presynaptic vesicular neurotransmitter transporters VGLUT1 and VGAT was also decreased in the tumor. Additionally, the fraction of parvalbumin and somatostatin nonneoplastic interneurons was reduced. In conclusion, changes in the levels of key proteins in neurotransmitter signaling suggest a loss of synapses and may thereby lead to neuronal network alterations in mouse GGs.

## Introduction

Gangliogliomas (GGs) are glioneuronal tumors representing the most common entity associated with temporal lobe epilepsy [1, 2, 3, 4]. Histologically, they are composed of dysmorphic neurons and neoplastic astroglial cells [1].

The functional relevance of dysmorphic neurons in GGs is not clear. Dysmorphic neurons have been occasionally found to be neuroactive in GGs [2, 3, 4]. Even though immunohistochemical analysis of MAP2 and NeuN proteins is useful to reveal the neuronal component in human GGs [5], there is no specific antigen marker to differentiate between preexisting and dysmorphic/tumorigenic neurons. Therefore, the recently developed in utero electroporation (IUE)-based GG mouse models represent a valuable tool to characterize the neuronal component in regard to cells harboring the oncogenes in a controlled experimental setting [4, 6].

Alterations in the levels of proteins involved in neurotransmitter signaling, so-called neurochemical molecules, and in the abundance of inhibitory neurons have been previously shown in human GGs [2]. In this context, glutamate and GABA receptors have been detected in the neuronal component of human GGs [3, 7]. Moreover, the fraction of parvalbumin interneurons has been found to be reduced in these tumors [2, 8].

However, a systematic neurochemical characterization clearly differentiating tumorigenic and preexisting neurons in the tumor tissue has so far not been performed due to technical challenges in human GG biopsies. Here, we used a new IUE-based mouse model for GGs in order to (1) assess the neuronal elements within GGs with regard to tumorigenic and preexisting cells and (2) characterize the neurochemical profile of the tumor compared to the control cortex at the protein level to assess changes in neurotransmitter-mediated signaling.

## Materials and Methods

### Mice

All animal experiments involving mice were conducted following the guidelines of the European Union and the University of Bonn Medical Center Animal Care Committee. To achieve a higher IUE efficiency, CD1/C57BL/6 hybrid mice were generated by crossing CD1 with B6.129P2-*Trp53*^*tm1Brn*^/J from Jackson Laboratories (Stock No. ‣008462). All animals were sacrificed at postnatal day 40. For the whole study, a total number of 6 mice were used.

### Intraventricular in utero Electroporation

The IUE approach was performed as previously described [6]. Coordinated breedings were performed for 1 day and the pregnant mothers were operated after 14 days. Mouse mothers received Ketoprofen and Buprenorphine 30 min before the surgery and isoflurane was given as a narcotic during the whole procedure. The solution containing the DNA plasmids at a final concentration of 1.5 μg/μL was mixed with Fast Green FCF to visualize the solution. After uterus exposure, DNA was injected into the lateral ventricle of the embryos by introducing a glass capillary pipette inside the cavity and expelling it by pressure with a microinjector. Electrical pulses of 30 V were delivered with the CUY21 SC Square Wave Electroporator. After surgery, females were kept on top of a heating pad and were subcutaneously injected daily with Ketoprofen for 3 days.

### Generation of Plasmids Used for IUE

The DNA plasmids injected with IUE were cloned into the PiggyBac (PB)-based vectors. In brief, the sequence of the transgenes was cloned between two PB terminal repeats in the donor PB plasmid (Wellcome Trust Sanger Institute, Cambridge, UK). *BRAF*^*V600E*^ transgene was kindly given by Dr. David Jones (German Cancer Research Center, Heidelberg) and *Akt*^*T308D/S473D*^ was purchased from Addgene (‣49192).

### Immunostaining

Mouse tumor brains were fixed in 10% buffered formalin for 7 days, embedded in paraffin, and sectioned at 4 μm. Formalin-fixed embedded tumor brains were then deparaffinized by incubation through a descending gradient of ethanol to water. For the heat-mediated antigen retrieval, we boiled the samples in citric buffer (pH 6.0, 10 mM) for 2 × 6 min. After a cooling period of 30 min, slices were washed with distilled water and PBS. To block nonspecific binding, sections were incubated with 10% normal goat serum and 1% fetal bovine serum in PBS for 2 h at 37°C. Each section was incubated with a primary antibody (online suppl. Table S1; for all online suppl. material, see www.karger.com/doi/10.1159/000528587) diluted in blocking buffer overnight at 4°C. For all slices, immunostaining against mCherry was performed to determine the localization of the tumor cells. The following day the sections were washed with distilled water and PBS and incubated with species-specific secondary goat antibodies conjugated to Alexa Fluor 488, 568, or 647 (Thermo Fisher Scientific; online suppl. Table S2) at a dilution of 1:200 in PBS containing 10% normal goat serum and 1% fetal bovine serum and DAPI was added with a dilution of 1:100. Finally, the sections were rinsed with PBS 2 × 5 min and then mounted with Mowiol.

### Confocal Imaging

All confocal images were captured using a Nikon Eclipse Ti Confocal Microscope. For every biological replicate, 5 images were taken from the tumor and control region. As a control, the cortical areas without IUE-positive tumor tissue were imaged from the same tumor brain slices (undergoing the same immunohistochemical procedure). Sections were acquired with a ×60 objective magnification, framing a 512 × 512 pixels image using a zoom of 2. Settings were kept the same between tumor and control areas (from the same brain slice) for each specific neurochemical marker (not necessarily applying the same settings for different *BRAF*^*V600E*^/*Akt*^*DD*^ biological replicates).

### Quantification

Acquired images were semiautomatically quantified and processed using the Fiji software offered by ImageJ. Somatic staining and co-labeling were analyzed with the JACoP and Cell Counter plugin of ImageJ. Nonsomatic stainings were automatically assessed by measuring the mean gray value. For control purposes, histoanatomically regularly structured neocortical areas within the same sections were used. These areas were clearly separated from the lesion-containing portions with the clear benefit of subjecting neoplastic and nonneoplastic areas from the same biological replicate to identical immunochemical conditions. This procedure has proved optimal for the evaluation of protein amount in brain developmental lesions associated with epilepsy in mouse and human tissue [36, 37].

### Statistics

All statistical analyses were performed by using Graph Prism software. All data are presented as means ± SEM.

## Results

### BRAF^V600E^/Akt^DD^ IUE Results in Glioneuronal Tumors with Features of Human GGs with Dysmorphic Neurons in a Glial Lesion Matrix

**Fig. 1:**
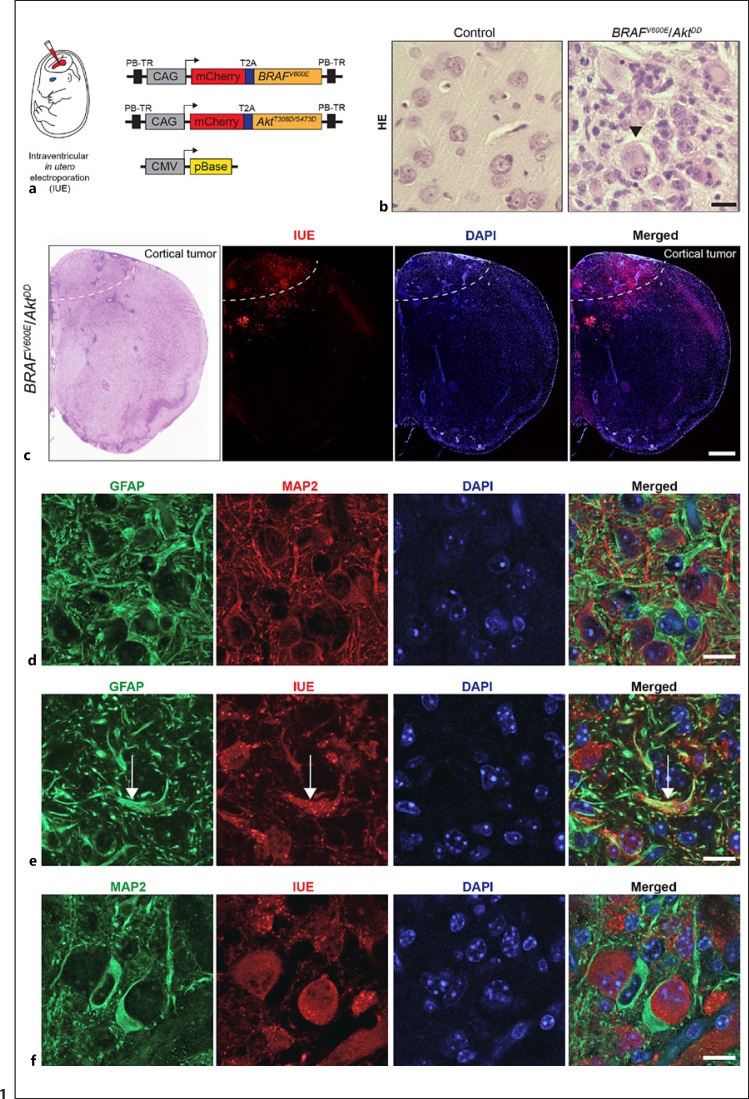
Immunohistological analyses of *BRAF*^*V600E*^/*Akt*^*DD*^ mouse tumors. **a** Schematic illustration of the PB plasmids and transposase used for IUE. Transgenes coding for *BRAF*^*V600E*^ and *Akt*^*T308D/S473D*^ (*Akt*^*DD*^) and a red fluorescent marker protein (mCherry) were cloned separated by the T2A peptide into the PB vector under the control of the ubiquitous CAG promoter and flanked by PB terminal repeats (PB-TR). The pBase transgene encoding for the PB transposase is expressed under the control of the constitutive CMV promoter. **b** High magnification image of hematoxylin and eosin (HE)-stained control cortex and *BRAF*^*V600E*^/*Akt*^*DD*^ tumor. Arrowhead points to a dysplastic neuron. Scale bar, 25 μm. **c** Overview images of a coronal *BRAF*^*V600E*^/*Akt*^*DD*^ mouse brain section stained with HE and corresponding expression of the IU-electroporated mCherry (referred to as IUE). The dashed line marks the border of the cortical tumor. Brains were isolated at postnatal day 40. Scale bar, 500 μm. **d** Immunostaining for GFAP and MAP2 protein of *BRAF*^*V600E*^/*Akt*^*DD*^ tumor showing the glioneuronal composition of the tissue. Scale bar, 25 μm. **e** High magnification fluorescence images of *BRAF*^*V600E*^/*Akt*^*DD*^ brain sections stained against GFAP and IUE marker. Note the positive staining of GFAP by IUE cells (arrow). Scale bar, 25 μm. **f** Fluorescence images of *BRAF*^*V600E*^/*Akt*^*DD*^ brain slices co-stained against MAP2 and IUE marker. Note the presence of IUE elements positive for MAP2. Scale bar, 25 μm.

In order to study the neurochemical profile of GGs, we developed a novel mouse model by targeting neural precursor cells at embryonic day 14 with intraventricular IUE. We injected DNA plasmids encoding BRAF^V600E^ and the constitutively active Akt form Akt^T308D/S473D^ (Akt^DD^), both in combination with a mCherry fluorescent tag (further named IUE) to track the IUE/tumor cells (Fig. 1a). The histological examination of the resulting tumors at postnatal day 40, further referred to as *BRAF*^*V600E*^/*Akt*^*DD*^, showed striking differences in tissue architecture between tumor (IUE-positive) and control (IUE-negative) cortex. The induced neoplasms were characterized by large dysplastic neurons entrapped within a distorted and dense neoplastic cell component and recapitulated thereby the striking features of human GGs (Fig. 1b). *BRAF*^*V600E*^/*Akt*^*DD*^ neoplasms were located within the cortex (Fig. 1c).

To verify the glioneuronal architecture, we first stained *BRAF*^*V600E*^/*Akt*^*DD*^ brain slices with antibodies against the glial fibrillary acidic protein (GFAP) and microtubule-associated protein 2 (MAP2) and found a strong immunoreactivity for both markers within the tumor area (Fig. 1d). To further verify the involvement of astrocytes within the tumor, we performed immunostaining against GFAP and the IUE marker and our results showed GFAP-positive labeling in IUE cells (Fig. 1e). Moreover, staining against MAP2 showed that many IUE cells, including large dysmorphic ones, were positive for this marker (Fig. 1f). Therefore, both GFAP and MAP2 cells were found to be targeted by the IUE approach within the tumor parenchyma.

### Quantitative and Qualitative Characterization of Neuronal *BRAF*^*V600E*^/*Akt*^*DD*^ Tumor Components

**Fig. 2:**
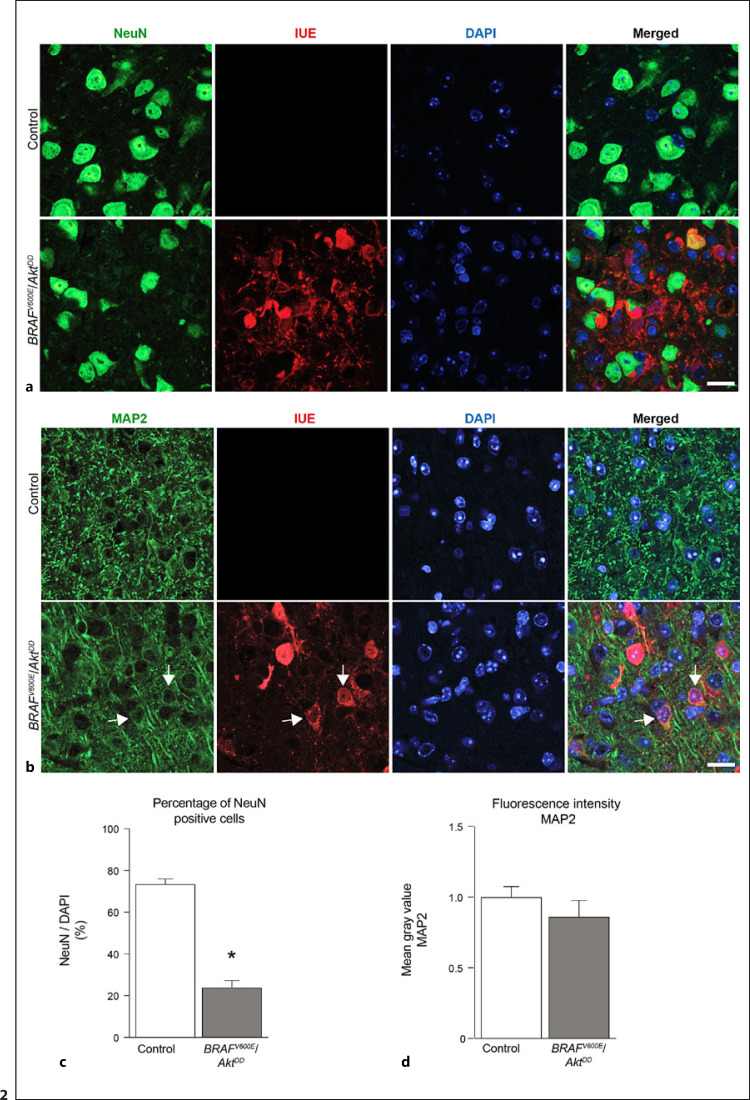
Comparison of NeuN- and MAP2-positive cells in control cortex and BRAFV600E/AktDD mouse tumors. **a** Immunolabeling of NeuN in the control and *BRAF*^*V600E*^/*Akt*^*DD*^ tumor tissue. IUE shows the injected cells. Note the presence of NeuN cells within the tumor tissue. Scale bar, 25 μm. **b** Immunofluorescence labeling of MAP2 in *BRAF*^*V600E*^/*Akt*^*DD*^ tumor and control regions. IUE depicts the targeted cells in the tumor area. Note the presence of MAP2-immunolabeled cells in both conditions. Scale bar, 25 μm. **c** Bar graph showing the percentage of NeuN-positive cells normalized to DAPI nuclei in the control and *BRAF*^*V600E*^/*Akt*^*DD*^ tumor tissue. Mann-Whitney test. *p < 0.05 (*n* = 4). **d** Normalized fluorescence intensity of MAP2-stained brain sections quantified by using the mean gray value. Mann-Whitney test. *p < 0.05 (*n* = 4).

Antibodies against NeuN and MAP2 were used to further characterize the neuron-like component in the control as well as *BRAF*^*V600E*^/*Akt*^*DD*^ tumor tissue. Immunostainings against NeuN showed the presence of NeuN-positive cells in both regions. Interestingly, most NeuN-positive cells were negative for mCherry expression in the tumor region (>90%, data not shown), indicating that the remaining NeuN-positive neurons within the neoplastic tissue were not hit by IUE (Fig. 2a). Further immunostaining against the somatodendritic marker MAP2 was used to depict the neuropil in the control and *BRAF*^*V600E*^/*Akt*^*DD*^ tumor tissue. MAP2-positive cells were found in both conditions, and as already pointed out, many MAP2-immunolabeled IUE cells were found within the tumor (Fig. 2b).

Quantitatively, the fraction of NeuN-immunoreactive cells (normalized to the number of DAPI-positive nuclei) in the *BRAF*^*V600E*^/*Akt*^*DD*^ tumors was significantly reduced in comparison to the control tissue (Fig. 2c). On the other hand, the quantification of the signal of MAP2 immunoreactivity did not show statistically significant changes between the *BRAF*^*V600E*^/*Akt*^*DD*^ tumor and control cortex (Fig. 2d). Because tumor tissue contains more cells than PTME, we also quantified MAP2 immunoreactivity by measuring the fluorescence intensity outside the DAPI-positive regions. Our results showed no differences between both quantifications (data not shown), assuming that the quantity of cells does not influence the levels of fluorescence intensity.

### Decrease of GABA_A_Rα1 but Not NMDAR1 Expression Levels in *BRAF*^*V600E*^/*Akt*^*DD*^-Induced GG-Like Tumors

Considering changes affecting the neuronal architecture between the control and tumor cortical areas, we next aimed to assess the abundance of key proteins involved in neurotransmitter signaling in these regions. Glutamate and GABA receptors are the most important postsynaptic receptors involved in excitatory and inhibitory neurotransmission, respectively.

**Fig. 3:**
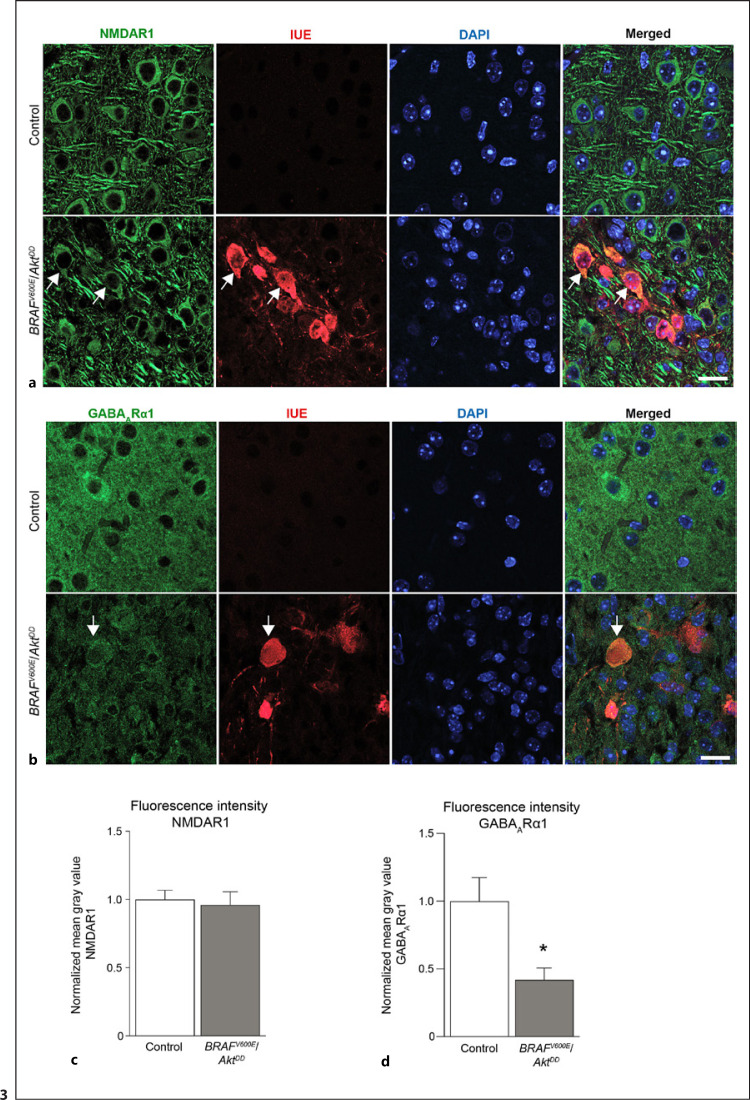
Immunochemical detection of NMDAR1 and GABA_A_Rα1 in control and tumor *BRAF*^*V600E*^/*Akt*^*DD*^ mouse brain cortex. **a** Representative images of control and tumor regions from *BRAF*^*V600E*^/*Akt*^*DD*^ brain sections immunostained with antibodies against NMDAR1 and IUE marker. Arrows in the lower panel point to NMDAR1 in IUE-positive cells. Scale bar, 25 μm. **b** Representative immunofluorescence images of GABA_A_Rα1 protein and IUE marker for control and *BRAF*^*V600E*^/*Akt*^*DD*^ tumor areas. Arrow points to an IUE cell positive for GABA_A_Rα1 protein (lower panel). Scale bar, 25 μm. **c** Quantification of the normalized fluorescence intensity levels of the NMDAR1 signal. **d** Bar graph showing the quantification of the normalized fluorescence intensity of GABA_A_Rα1 labeling for the control and *BRAF*^*V600E*^/*Akt*^*DD*^ regions. The fluorescence intensity is plotted as the normalized mean gray value of the fluorescent signal. Mann-Whitney test. *p < 0.05 (*n* = 4).

Therefore, we first analyzed the abundance of the glutamatergic *N*-methyl-D-aspartate receptor 1 (NMDAR1). Our results showed the presence of NMDAR1 in both control and *BRAF*^*V600E*^/*Akt*^*DD*^ tumor cortex. Moreover, we observed that some of the IUE tumor cells expressed this receptor (Fig. 3a). Furthermore, we characterized the protein levels of the gamma-aminobutyric acid A receptor alpha 1 (GABA_A_Rα1). We also found immunoreactivity against this postsynaptic receptor in some IUE cells (Fig. 3b).

Quantitatively, we did not observe changes in the fluorescence intensity in NMDAR1-stained sections between both groups (Fig. 3c). In contrast to NMDAR1, immunolabeling against GABA_A_Rα1 revealed a decrease in the abundance of this receptor in the *BRAF*^*V600E*^/*Akt*^*DD*^ tumor compartment. The corresponding quantification of the fluorescence intensity showed a significant reduction of the fluorescence intensity in the *BRAF*^*V600E*^/*Akt*^*DD*^ tumor versus the control cortex (Fig. 3d).

Overall, these data indicated quantitative alterations in the GABA_A_Rα1 protein abundance within the tumor area, whereas levels of the glutamatergic NMDAR1 were unchanged. Interestingly, we found expression of both postsynaptic receptors in or in close proximity to some IUE cells.

### Reduced Protein Levels of the Presynaptic Vesicular Neurotransmitter Transporters VGLUT1 and VGAT within the *BRAF*^*V600E*^/*Akt*^*DD*^ Tumor Tissue

**Fig. 4:**
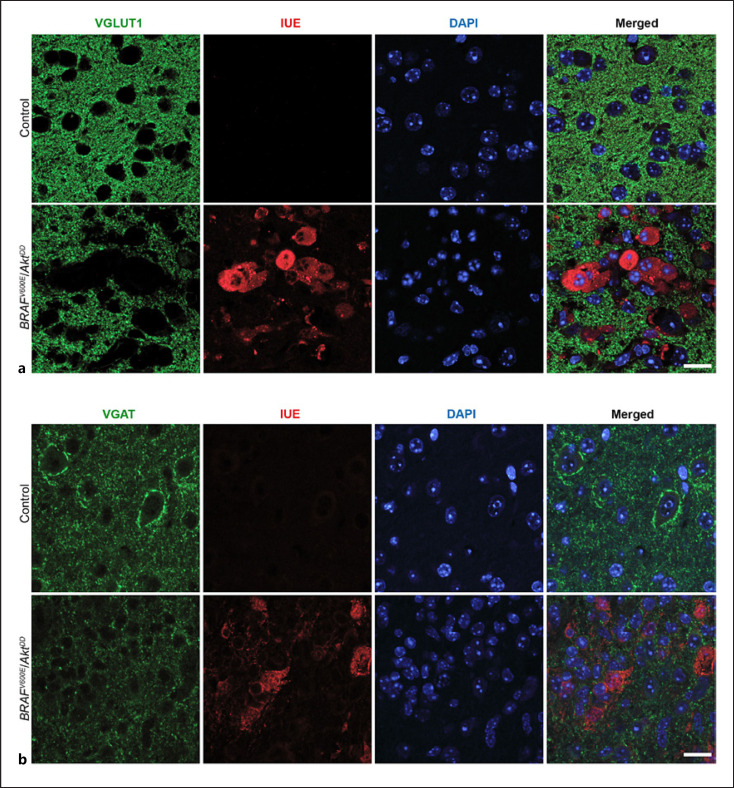
Immunochemistry for VGLUT1 and VGAT presynaptic transporters in the control and tumor cortex of *BRAF*^*V600E*^/*Akt*^*DD*^ brains. **a** Representative immunofluorescence images showing VGLUT1 expression in control and *BRAF*^*V600E*^/*Akt*^*DD*^ tumor cortex. Scale bar, 25 μm. **b** Images showing immunostaining for VGAT in control and *BRAF*^*V600E*^/*Akt*^*DD*^ tumor cortex. Scale bar, 25 μm.

We next wanted to characterize the vesicular glutamate transporter 1 (VGLUT1) and the vesicular GABA transporter (VGAT), both located in the membrane of presynaptic vesicles. As expected, VGLUT1 immunolabeling in the control cortex revealed a punctate pattern but was not observed in the soma of cells indicative of a synaptic localization (Fig. 4a, upper panels). However, the cortical areas containing tumor cells were devoid of fluorescence derived from VGLUT1. The immunostaining results showed no VGLUT signal in individual tumor cells and revealed the presence of VGLUT synapses surrounding tumor cell clusters (Fig. 4a, lower panels).

Immunostaining against VGAT showed the presence of the protein in both the control and tumor cortex. However, the pattern of the fluorescent signal from the staining differed between the conditions. While in the control we found strong perisomatic immunolabeling for VGAT in cortical cells revealing the inhibitory synapses, the tumor cells did not show VGAT immunofluorescence on the surface of their soma (Fig. 4b). These observations from the immunochemical results suggest a redistribution of presynaptic nerve terminals in the tumor.

### Low Parvalbumin- and Somatostatin-Positive Interneuron Density in *BRAF*^*V600E*^/*Akt*^*DD*^ Tumors

We further wanted to assess the presence and distribution of interneurons within the tumor compared to the control cortical region. For this purpose, we stained *BRAF*^*V600E*^/*Akt*^*DD*^ brain slices with antibodies against parvalbumin and somatostatin, two markers for different interneuron subtypes. Since we reported that the induced mouse tumors have a reduction in the percentage of NeuN-positive cells per se (Fig. 2c), we included the co-staining of NeuN and quantified the percentage of parvalbumin and somatostatin neurons among the NeuN-positive cells.

**Fig. 5:**
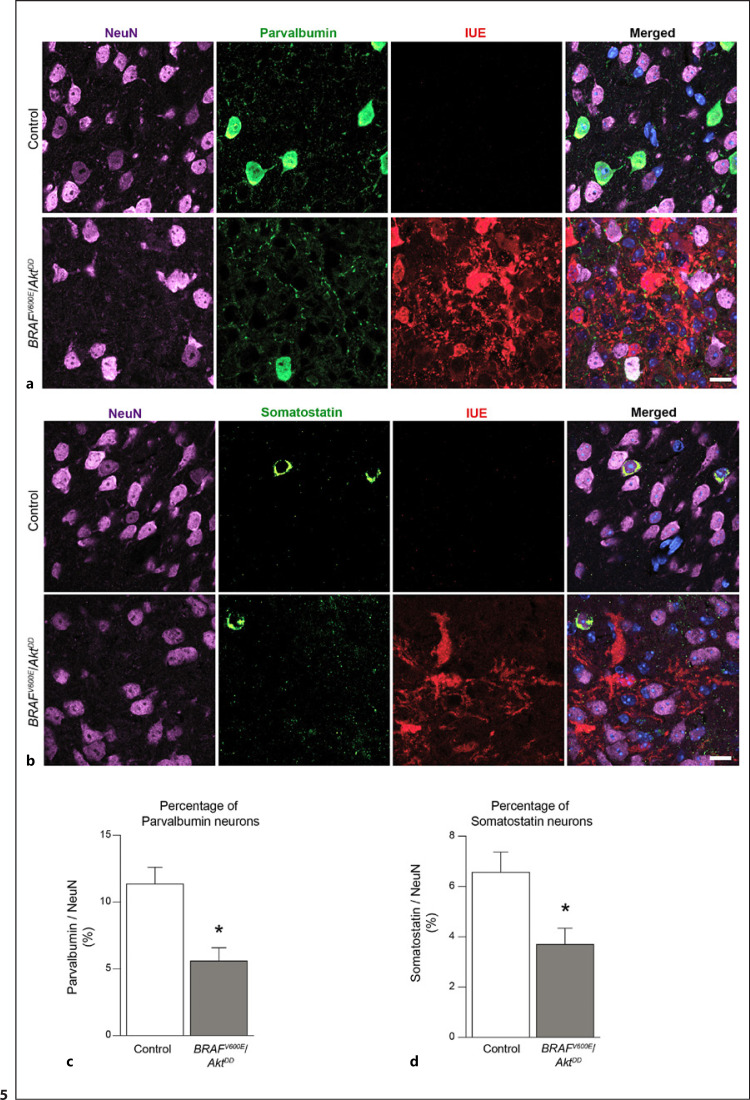
Distribution of parvalbumin- and somatostatin-positive neurons in control and *BRAF*^*V600E*^/*Akt*^*DD*^ tumor cortical regions. **a** Images from *BRAF*^*V600E*^/*Akt*^*DD*^ brain sections co-stained against NeuN, parvalbumin and IUE marker for control and tumor. NeuN is used as a marker for neuronal elements. **b** Representative images are shown for immunofluorescence staining against NeuN, somatostatin, and IUE protein in control and *BRAF*^*V600E*^/*Akt*^*DD*^ tumor cortical regions. Scale bar, 25 μm. **c** Bar graph showing the quantification of the percentage of parvalbumin-positive cells among the total amount of NeuN-labeled cells. Scale bar, 25 μm. **d** Corresponding quantification of the percentage of cells positively labeled with somatostatin among the total amount of NeuN-positive cells. Mann-Whitney test. *p < 0.05 (*n* = 4–6).

Our results showed a significant reduction in the fraction of parvalbumin-positive neurons in the *BRAF*^*V600E*^/*Akt*^*DD*^ tumors compared to the control (Fig. 5a, c). Moreover, the number of neurons with immunoreactivity against somatostatin was significantly reduced as well (Fig. 5b, d). To further assess the extent of this reduction, we quantified the percentage of interneurons in the peritumoral region. The corresponding results demonstrated that the fractions of parvalbumin-expressing cells in this area and the tumor parenchyma itself were not significantly different (online suppl. Fig. S1a, c). Intriguingly, the somatostatin-positive cells were significantly more abundant in the perilesional tissue compared to the tumor parenchyma (online suppl. Fig. S1b, d). These results suggest different relative abundances of different interneuronal cell types in peritumoral regions relative to the glioneuronal lesion areas.

Co-immunohistochemistry with antibodies against mCherry showed that none of the interneurons was positive for IUE. Taken together, these results indicated that the *BRAF*^*V600E*^/*Akt*^*DD*^ tumor parenchyma comprises lower fractions of parvalbumin- and somatostatin-positive interneurons.

## Discussion

The present analyses revealed striking neurochemical profile patterns in murine GGs induced by IUE of *BRAF*^*V600E*^ and *Akt*^*DD*^. The genetic architecture of the GG mouse model used in the present study has been reasoned based on molecular-genetic observations in human GGs, in which *BRAF*^*V600E*^ is the most common mutation detected in 45–60% of human tumors [9, 10, 11]. In parallel, the activation of mTOR signaling pathway is a robust observation in human GGs [12, 13]. Going beyond the potential of immunohistochemical analyses in human GGs [2], the use of the mouse GG model for our analyses allows a clear separation between tumor and microenvironmental compartments and cells. Our study showed reduced levels of GABA_A_Rα1 in the tumor compared to nontumoral brain tissue, which is in agreement with previous reports, showing that the expression of GABA receptors is downregulated in human GGs [14, 15]. Despite the protein levels of GABA_A_Rα1 being reduced in the *BRAF*^*V600E*^/*Akt*^*DD*^ tumor tissue, the receptor was still expressed by IUE/tumor cells.

On the other hand, NMDAR1 protein levels in the *BRAF*^*V600E*^/*Akt*^*DD*^ tumors were unchanged compared to the regular cortex. Indeed, dysmorphic neurons in human GG express NMDAR1 [2, 3, 16] and we further demonstrated in the mouse model that the receptor is expressed by IUE/tumor cells. Overall, a decrease of GABA_A_Rα1s in concert with unaltered NMDAR1 protein levels may have an impact on the excitation/inhibition balance in mouse GGs. Altered subcellular distribution of the molecules under study may represent an additional level of complexity predisposing to altered excitability due to GGs. However, the fluorescence intensity assessed from the whole image captured postsynaptic receptors located in both somatic and dendritic compartments. The fundamental structural and morphological alterations of neurites of dysmorphic GG neuronal elements render a comparative analysis of clearly defined cellular subcompartments virtually impossible by the presently used approaches, such that the complexity of alterations of the molecules under study in GGs may be even underestimated.

The potential relevance of NMDAR1 with respect to altered excitability related to glioneuronal tumors is underlined by the fact that it is the functional subunit of the receptor [17, 18] and has been described as being expressed in human GGs from patients with chronic intractable epilepsy [19]. For the alpha 1 subunit of the GABA_A_R, it has been previously suggested to contribute to the pathophysiology of seizures [20, 21]. For instance, viral delivery of the subunit has been shown to reduce the incidence of spontaneous seizures [22]. Moreover, a decreased expression of the alpha 1 subunit has been observed after status epilepticus in adult rodents [23]. Future work will need to address whether additional GABA and NMDA receptor subunits are altered in expression in GGs and have a potential impact on GG-related network hyperexcitability.

Besides alterations in the postsynaptic compartment, the levels of VGLUT1 presynaptic glutamate transporter were found to be reduced in the *BRAF*^*V600E*^/*Akt*^*DD*^ tumor compared to the regular structured cortex. A similar labeling pattern was observed for the presynaptic GABA transporter, VGAT whose protein levels were reduced in the *BRAF*^*V600E*^/*Akt*^*DD*^ tumor. Considering the cell composition of these tumors, we cannot exclude that the presence of a dense neoplastic glial component may lead to a reduction of the overall density of presynaptic contacts. Moreover, it is worth noticing that excitatory and inhibitory synapses may not be equally affected; we did not observe changes in the NMDAR1 protein levels, but a robust reduction in VGLUT1 in the tumor compared to the PTME. In contrast, the reduction observed in GABA_A_Rα1 was much higher than the decrease affecting VGAT. These alterations could lead to changes in extracellular levels of glutamate and GABA causing E/I imbalance.

A reduction in parvalbumin and somatostatin cells has been reported in human GG tissues [2, 7, 8], and this phenomenon was suggested to trigger hyperexcitability in an animal model of cortical dysplasia [24]. Our data demonstrated a reduction of the fraction of parvalbumin- and somatostatin-expressing interneurons in the *BRAF*^*V600E*^/*Akt*^*DD*^ tumor parenchyma also in the GG mouse model. Parvalbumin- and somatostatin-positive interneurons derive from the medial ganglionic eminence and migrate tangentially to the developing cortex [25, 26, 27, 28]. The IUE approach only targets neural precursor cells at the ventricular surface that migrate radially to occupy the cerebral cortex during brain development [29]. It is therefore not surprising that parvalbumin- and somatostatin-expressing cells present in the tumor are negative for IUE and prompts the conclusion that the reduction in the percentage of parvalbumin- and somatostatin-expressing cells is not directly mediated by the introduction of genetic alterations.

In fact, this long tangential migration path is strictly regulated and controlled by chemoattractants [30, 31], including neurotrophic factors and neurotransmitters. Thus, changes in the extracellular levels of GABA and glutamate can importantly interfere with the migration of the interneurons into the cortex during development [32, 33]. The fact that interneurons are still migrating to their target layer after cells derived from ventricular progenitors have been positioned in their cortical regions [28] may suggest the following hypothetic scenario: IUE of *BRAF*^*V600E*^ and *Akt*^*DD*^ plasmids into neural precursor cells triggers tumor development, which may cause architectural and chemoattractive changes in the cortex providing a less optimal local microenvironment for the interneurons to invade the neoplastic lesion area. Moreover, considering the differences observed in the peritumoral region and also the fact that the migration of somatostatin and parvalbumin is controlled by different signaling pathways during brain development [34], its final position may be also controlled by different effects from tumor tissue. At this point, we cannot entirely rule out that a reduced expression of parvalbumin and somatostatin takes place in cellular subcompartments of interneurons within the GGs without loss of the cells themselves − similar to what has been suggested in epilepsy-associated hippocampal sclerosis [35]. Despite the fact that due to the irregular neurite structure within GGs, it may be doubtful whether the same alterations with respect to loss of parvalbumin in the somatodendritic compartment and partly the axon occur in neurodevelopmental lesions given by GGs in this case as in neurodegeneration inducing insult conditions given by hippocampal sclerosis. Considering that interneurons are also important to keep the balance between E/I, a decrease in their percentage could provide another layer of complexity affecting the overall neuronal network activity.

We conclude that GG tumor development leads to changes in the number and distribution of synaptic contacts and excitatory and inhibitory neurons might not be altered to a similar degree, thereby possibly causing excitation/inhibition imbalance and changes to neuronal network activity.

## Statement of Ethics

We thank the Bonn Medical Faculty for providing the ethical framework of the Epilepsy-surgical Biobank. The protocols for the animal experiments were reviewed and approved by Landesamt für Natur, Umwelt und Verbraucherschutz Nordrhein-Westfalen (LANUV) under the permission number 81-02.04.2019.A030.

## Conflict of Interest Statement

None of the authors have a conflict of interest.

## Funding Sources

Our work is supported by Deutsche Forschungsgemeinschaft SFB 1089 (TP D10, D-259.0454) and FOR 2715 (BE 2078/10-2) to A.J.B.; SFB1089: TP A06, P03, SCHO 820/4-7, SPP1757 to S.S.), CONNECT-GENERATE (FKZ01GM1908C to A.J.B.) as well as MSSO (SciMed grant in aid to P.M.) and the Else Kröner-Fresenius Stiftung (“Neuroimmunology” BonnNi grant in aid to M.K.).

## Author Contributions

M.K. and S.C.C. directed, performed, and guided all major parts of the manuscript and experimental work. Furthermore, J.P., K.M.J.v.L., S.S., and A.J.B. contributed to the conception and design of the study; P.M., A.Q., and A.J.B. provided immunohistochemical and neuropathological expertise; T.O. and S.S. contributed significantly to the interpretation of the data; M.K. and S.C.C. wrote the initial complete version of the manuscript and provided all figures; and all authors edited the manuscript for further intellectual content and approved its final version.

## Data Availability Statement

All data that support the findings of this study are included in this article and its online supplementary material. Further inquiries can be addressed to the corresponding author.

## Supplementary Material

Supplementary data
