# Supplementary material for: Neurochemical Profile of BRAFV600E/AktT308D/S473D Mouse Gangliogliomas Reveals Impaired GABAergic System Inhibition
Source: Dev Neurosci. 2022 Dec 20;45(2):53–65. doi: 10.1159/000528587 (PMC10129025; doi:10.1159/000528587)
Supplement: Supplementary file 1 — Supplementary data [file dne-0045-0053-s01.docx]

Neurochemical profile of *BRAF^V600E^*/*Akt^T308D/S473D^* mouse gangliogliomas reveals impaired GABAergic system inhibition

Maria Kyriazi^a^, Philipp Müller^a^, Julika Pitsch^b^, Karen M. J. van Loo^c^, Anne Quatraccioni^a^, Thoralf Opitz^d^, Susanne Schoch^a,b^, Albert J. Becker^a^ and Silvia Cases-Cunillera^a^*

^a^Institute of Neuropathology, Section for Translational Epilepsy Research, Medical Faculty, University of Bonn, 53127 Bonn, Germany;

^b^Department of Epileptology, Medical Faculty, University of Bonn, 53127 Bonn, Germany;

^c^Department of Epileptology, Neurology, RWTH Aachen University, 52074 Aachen, Germany;

^d^Institute of Experimental Epileptology and Cognition Research, Medical Faculty, University of Bonn, 53121 Bonn, Germany.

***Corresponding author:**

Silvia Cases-Cunillera, PhD

Section for Translational Epilepsy Research, Department of Neuropathology

University of Bonn Medical Center

Sigmund-Freud Str. 25

DE-53105 Bonn (Germany)

Tel: +49-228-287-19346

Email: [silviacases@uni-bonn.de](mailto:silviacases@uni-bonn.de)

**Supplementary material**

**
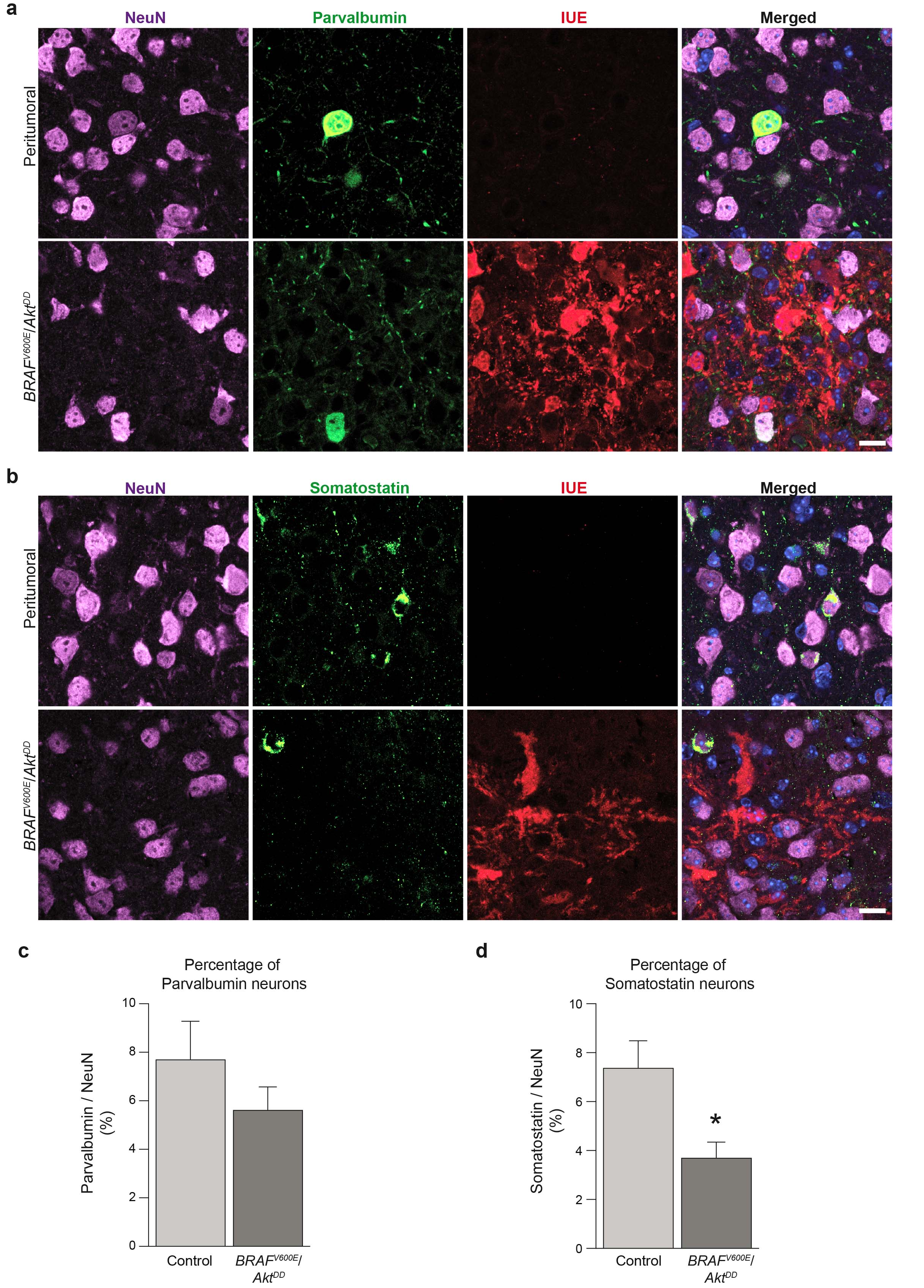
**

**Supplementary Fig. S1. Distribution of parvalbumin- and somatostatin-positive neurons in peritumoral and *BRAF^V600E^*/*Akt^DD^* tumor cortical regions. a**. Images from immunochemical stainings of the peritumoral and tumor regions from *BRAF^V600E^*/*Akt^DD^* brain slices with antibodies against parvalbumin and NeuN. Scale bar, 25 μm. **b**. Co-immunostaining images for somatostatin and NeuN in the peritumoral and tumor regions from *BRAF^V600E^*/*Akt^DD^* brain slices. Scale bar, 25 μm. **c**. Bar graph with the quantification of the percentage of parvalbumin neurons within the peritumoral and tumor regions. **d.** Quantification of the fraction of somatostatin-positive neurons in the peritumoral compared to tumor regions. Mann-Whitney test. *p < 0.05 (n = 4 - 6).

**Table S1.** List of the primary antibodies used for the immunochemical analysis.

| **Primary Antibodies** | **Dilution** | **Source** | **Catalog #** |
| --- | --- | --- | --- |
| mCherry | 1:200 | Abcam | ab167453 |
| mCherry | 1:200 | Abcam | ab125096 |
| GFAP | 1:400 | Sigma Aldrich | G3893 |
| NeuN | 1:200 | Synaptic Systems | 266004 |
| MAP2 | 1:200 | Millipore | MAB3418 |
| NMDAR1 | 1:100 | Abcam | ab17345 |
| GABA_A_Rα1 | 1:1000 | Synaptic Systems | 224203 |
| vGlut1 | 1:200 | Synaptic Systems | 135304 |
| VGAT | 1:100 | Synaptic Systems | 131 308 |
| Parvalbumin | 1:2000 | Sigma Aldrich | P3088 |
| Somatostatin | 1:100 | Millipore | MAB354 |

**Table S2.** List of secondary antibodies used for immunochemistry.

| **Secondary Antibodies** | **Dilution** | **Source** | **Catalog #** |
| --- | --- | --- | --- |
| Alexa Fluor 488 Goat anti-Mouse IgG (H+L) | 1:200 | Thermo Fisher Scientific | A-11001 |
| Alexa Fluor 568 Goat anti-Mouse IgG (H+L) | 1:200 | Thermo Fisher Scientific | A-11004 |
| Alexa Fluor 647 Goat anti-  Rat IgG (H+L) | 1:200 | Thermo Fisher Scientific | A-21247 |
| Alexa Fluor 647 Goat anti-Guinea Pig IgG (H+L) | 1:200 | Thermo Fisher Scientific | A-21450 |
| Alexa Fluor 488 Goat anti-Guinea Pig IgG (H+L) | 1:200 | Thermo Fisher Scientific | A-11073 |
| Alexa Fluor 488 Goat anti-Rabbit IgG (H+L) | 1:200 | Thermo Fisher Scientific | A-11008 |
| Alexa Fluor 568 Goat anti-Rabbit IgG (H+L) | 1:200 | Thermo Fisher Scientific | A-11011 |
